# High-Density Lipoproteins and Serum Amyloid A (SAA)

**DOI:** 10.1007/s11883-020-00901-4

**Published:** 2021-01-15

**Authors:** Nancy R. Webb

**Affiliations:** Department of Pharmacology and Nutritional Sciences, Saha Cardiovascular Research Center, and Barnstable Brown Diabetes Center, University of Kentucky, 553 Wethington Building, 900 South Limestone, Lexington, KY 40536-0200 USA

**Keywords:** Acute phase response, Atherosclerosis, HDL remodeling, Inflammation, Innate immunity

## Abstract

**Purpose of Review:**

Serum amyloid A (SAA) is a highly sensitive acute phase reactant that has been linked to a number of chronic inflammatory diseases. During a systemic inflammatory response, liver-derived SAA is primarily found on high-density lipoprotein (HDL). The purpose of this review is to discuss recent literature addressing the pathophysiological functions of SAA and the significance of its association with HDL.

**Recent Findings:**

Studies in gene-targeted mice establish that SAA contributes to atherosclerosis and some metastatic cancers. Accumulating evidence indicates that the lipidation state of SAA profoundly affects its bioactivities, with lipid-poor, but not HDL-associated, SAA capable of inducing inflammatory responses in vitro and in vivo. Factors that modulate the equilibrium between lipid-free and HDL-associated SAA have been identified.

**Summary:**

HDL may serve to limit SAA’s bioactivities in vivo. Understanding the factors leading to the release of systemic SAA from HDL may provide insights into chronic disease mechanisms.

## Introduction

Serum amyloid A (SAA) comprises a family of low molecular weight proteins (104–112 amino acid residues) first described almost 50 years ago (for a recent comprehensive review, see [1]). SAA was identified as the circulating protein that forms amyloid deposits in tissues of certain individuals suffering from chronic or recurrent inflammation [2, 3]. Although a rare disorder, secondary amyloidosis can cause severe clinical complications, such as kidney failure. While its physiological function(s) remain an enigma, SAA is remarkably conserved through millions of years of evolution, suggesting it plays an important role in primordial host defense. The human genome encodes two acute phase SAA proteins, SAA1 and SAA2, which are 96% homologous over their entire length and correspond to mouse SAA1.1 and SAA2.1. Mice encode a third conserved acute phase SAA gene, *Saa3*. *Saa3* is generally thought to be a pseudogene in humans due to an early stop codon [4]. In addition to the acute phase isoforms, humans and mice also encode another gene family member, *Saa4*. This isoform contains an insertion of 8 amino acids between residues 69 and 70 of SAA1/SAA1.1 and SAA2/SAA2.1. While the SAA4 protein is expressed in the liver at low levels and is not induced during inflammation, it likely comprises the major SAA isoform in the circulation under basal conditions [5, 6]. The constitutive SAAs are generally understudied members of the gene family and will not be covered in this review, which focuses on liver-derived acute phase SAAs.

In both humans and mice, the acute phase SAAs are transcriptionally regulated in hepatocytes by a variety of inflammatory cytokines including tumor necrosis factor-α, interleukin-1β, interleukin-6, and interferon-γ, which are all likely produced by macrophages during an acute inflammatory response (reviewed in [7]). SAA can transiently increase > 1000-fold in the circulation and, as such, is considered one of the most highly induced acute phase reactants in vertebrates. Indeed, it has been estimated that SAA represents ~2.5% of total hepatic protein synthesized in mice during endotoxemic shock [8]. There is also evidence that hepatic SAA production may be regulated through posttranscriptional mechanisms [9, 10]. Notably, SAA appears to be not only a key soluble mediator in the acute phase response but also likely plays a role in a negative feedback loop that serves to shut down inflammation [11].

## SAA and Chronic Inflammatory Disease

Given its profound induction and evolutionary conservation, acute phase SAAs likely play a key role in survival during traumatic injury or acute infection. Circulating SAA is also known to be persistently elevated, albeit at lower levels, in individuals with chronic inflammation. This “inappropriate” expression of SAA has been associated with increased risk or poor prognosis for numerous chronic diseases, including atherosclerotic cardiovascular disease and cancer [12, 13]. Whether SAA plays a direct role in the pathogenesis of these chronic diseases, or is merely a marker of increased risk, has been the topic of intense investigations over the past several decades. One obstacle has been the lack of knockout mice deficient in all three acute phase SAAs. The development of SAA-deficient mice was challenging, due to the length of the gene cluster encoding the three acute phase SAAs (~45 kb) on mouse chromosome 7 [14], which made it difficult to target all three genes simultaneously by conventional homologous recombination approaches. With the advent of CRISPR-Cas9 technology, mice deficient in SAA1.1, SAA2.1, and SAA3 have recently been generated [15, 16••].

It is well-recognized that elevated SAA is associated with increased risk for atherosclerosis in humans [17^•^]. In some reports, plasma SAA levels were a better predictor of future cardiovascular events than the widely used clinical biomarker of inflammation, hsCRP [18–20]. It has recently been suggested that SAA contributes to atherosclerosis and its complications at least in part through its prothrombotic effects [21^•^]. Several gain-of-function studies in mice using viral vector-mediated SAA overexpression demonstrated that SAA contributes to atherosclerotic processes and is not merely a biomarker reflecting the burden of the disease [22, 23]. Paradoxically, an early study by our group investigating the role of endogenous SAA in atherosclerotic lipid deposition showed no reduction in APOE^−/−^ mice that were deficient in SAA1.1 and SAA2.1 compared to control APOE^−/−^ mice [24]. However, in a follow-up study, we determined that suppression of SAA3 expression in APOE^−/−^ mice lacking SAA1.1 and SAA2.1 had significantly reduced atherosclerosis compared to APOE^−/−^ mice expressing all three SAAs [15], highlighting that SAA1.1, SAA2.1, and SAA3 likely play redundant roles in atherogenesis and that deficiency/suppression of all three acute phase isoforms is necessary to reduce atherosclerosis in mice. On the other hand, our group has shown that deficiency of SAA1.1 and SAA2.1 is sufficient to protect APOE^−/−^ mice from angiotensin II-induced abdominal aortic aneurysms [25]. Taken together, human epidemiological data and results from genetically altered mice support a causal role for SAA in cardiovascular disease, although the precise mechanisms remain unclear.

While dozens of publications spanning more than three decades report an association between circulating SAA levels and a spectrum of neoplastic diseases in humans, direct evidence that SAA promotes tumorigenic processes has been limited. A number of years ago, Sung et al. reported that forced overexpression of SAA in Lewis lung carcinoma cells increases the cells’ capacity to colonize the lung and form tumors in mice, indicating that SAA may be a tumor-intrinsic factor that promotes tumor cell metastasis [26]. A more recent study by Beatty and colleagues demonstrated for the first time that inflammatory responses mounted by hepatocytes are critical for the establishment of a metastatic niche in the liver, with SAA playing a key role [27^••^]. In a mouse model of pancreatic ductal adenocarcinoma, the authors showed that pancreatic tumors activate IL-6/STAT3 signaling in hepatocytes, leading to the induction of SAA, which in turn orchestrates local changes in the inflammatory and extracellular matrix milieu that supports tumor cell spread to the liver. Thus, current data suggest that targeting SAA may provide a novel strategy for preventing tumor metastasis to the liver.

## SAA as an HDL Apolipoprotein

In the blood, the vast majority (~95%) of liver-derived SAA is typically found associated with the high-density lipoprotein (HDL) fraction [28, 29, 30••]. During a severe inflammatory response, SAA can become the major apolipoprotein on HDL [31]. The SAA monomer is predicted to comprise four α helices arranged in a cone-shaped array [32], with helices 1 and 3 containing both hydrophobic and hydrophilic faces. According to one model, SAA assumes a topology that allows it to act as a “hub” in macromolecular interactions by associating with the lipid surface of the HDL particle as well as cellular receptors and extracellular components [33]. Extensive research has focused on whether the presence of SAA impacts HDL function during inflammation. Of particular interest is whether SAA alters HDL’s ability to mediate reverse cholesterol transport (RCT), the pathway by which excess cholesterol in peripheral tissues is delivered to the liver for excretion in bile and feces, and a major mechanism by which HDL is thought to be cardioprotective. Over the years, results from studies investigating the impact of SAA on HDL-mediated RCT have been conflicting. In a number of studies, SAA, either associated with HDL or in a lipid-free form, was reported to promote cellular cholesterol efflux through both ABCA1-dependent and ABCA1-independent mechanisms [34–37]. On the other hand, HDL isolated from human subjects undergoing acute sepsis [38] or experimental endotoxemia [39] showed a reduced capacity to promote cholesterol efflux, suggesting that SAA may impede RCT. According to several reports, fecal excretion of macrophage-derived cholesterol is reduced during inflammation in mice [38–40]. However, our studies in SAA-deficient mice suggest that this impairment of macrophage-to-feces RCT during inflammation is not dependent on SAA [40].

It has been suggested that SAA reduces the anti-inflammatory and antioxidative properties of HDL and thus renders HDL dysfunctional [41, 42•, 43•]. The extent to which an impairment in the anti-inflammatory or antioxidant activity of HDL contributes to chronic inflammatory disease is not clear; however, it is notable that HDL contributes only a minor portion (1–2%) of the total antioxidant capacity of plasma [44]. Although the antioxidative capacity of HDL has been shown to be reduced in a number of disease states (reviewed in [45]), it does not appear that SAA plays a direct role in this impairment. Jayaraman et al. [46] demonstrated that SAA enrichment of HDL actually impedes lipoprotein oxidation and that mild oxidation of SAA-enriched HDL leads to the release of SAA that exhibits antioxidant effects. This study, together with the work of Sato et al. [47], indicates that SAA does not produce a prooxidant effect on lipoproteins and may have antioxidant effects. Further support for an antioxidant effect of SAA is provided by a population study indicating that HDLs from patients with higher SAA levels exhibit enhanced antioxidant activity compared to controls [48]. In summary, while it has been extensively documented through the use of a variety of in vitro assays that HDL function is impaired in inflammation, a role for SAA in mediating HDL dysfunction in vivo is not well substantiated. Based on current evidence, it seems unlikely that the massive enrichment of HDL with SAA during an acute inflammatory response has evolved for the purpose of altering HDL function. Rather, the association of systemic SAA with HDL likely serves as a mechanism for increasing SAA stability [49^•^], or as discussed below, dampening SAA’s pro-inflammatory activities in the circulation.

## SAA as an Innate Immune Molecule

Given the accumulating evidence that SAA plays a causative role in a variety of chronic inflammatory diseases, there is a critical need to understand SAA’s biological functions. As summarized in a recent review [50], SAA has been shown to evoke a variety of activities consistent with its putative role as an innate immune molecule, including cytokine induction, leukocyte chemotaxis, and upregulation of genes involved in the remodeling of the extracellular matrix, including TGF-β and matrix metalloproteinases. These activities have been attributed to signaling through a number of “pattern recognition receptors” (PRRs) including formyl peptide receptor-like 1 (FPRL-1), FPRL-2, TLR2, TLR4, SR-BI, and CD36 [50]. More recently, SAA was shown to stimulate secretion of IL-1β by human and mouse macrophages by activating the NLRP3 inflammasome [51^•^]. However, it should be noted that many of the reports on SAA’s activities in vitro are unfortunately confounded by the recent recognition that a widely used, commercially available hybrid form of SAA exerts activities not shared by native SAA [52–55]. Thus, results from some earlier studies investigating the pro-inflammatory functions of SAA and its role in innate immunity must be interpreted with caution. Nevertheless, a preponderance of in vitro data supports the conclusion that SAA plays a key role in innate immunity by stimulating multiple inflammatory pathways during acute infection or tissue injury. In the setting of chronic inflammation, SAA may contribute to pathological processes by inappropriately activating inflammatory signaling.

A puzzling aspect of SAA biology is that the robust effects of SAA reported from in vitro studies do not appear to be easily recapitulated in vivo [53, 56]. This paradox is perhaps most clearly demonstrated by the work of Simons and colleagues, who developed transgenic mice with liver-specific, doxycycline-inducible expression of SAA [56]. Interestingly, when treated with doxycline, the transgenic mice failed to mount a systemic inflammatory response, despite a remarkable induction of SAA to plasma levels that correspond to a robust acute phase response (i.e., > 1 mg/ml). On the other hand, Chami et al. reported that administering SAA by i.p. injection produced a prothrombotic and pro-inflammatory phenotype in APOE^−/−^ mice that was accompanied by indices of renal dysfunction [57^•^]. However, in a subsequent study, the same group demonstrated that SAA’s pathological effects were ameliorated when the injected SAA was supplemented with HDL [58^••^]. All of these findings raise important questions about the mechanisms that influence SAA activities in vivo. To protect the host from tissue damage under homeostatic conditions, it seems likely there are mechanisms to blunt the inflammatory effects of circulating SAA unless it is present in the appropriate context. Without such mechanisms, profound induction of SAA during a robust acute phase response would be expected to lead to unrestrained tissue damage throughout the body, rather than targeted inflammation at the site of tissue injury or infection. As discussed above, under normal circumstances, virtually all of SAA in plasma is associated with HDL. Moreover, most in vitro studies have examined the effect of lipid-free SAA, not HDL-associated SAA, which may be a limitation since emerging literature indicates that many of the effects attributed to SAA are lost when SAA is HDL-bound [51•, 53, 59, 60]. Thus, the relationship of SAA with HDL must be taken into account when considering SAA’s pathophysiological effects in vivo.

## Generation of Non-HDL-Associated SAA

As noted above, recent findings clearly establish that lipid-free and HDL-associated SAA are functionally distinct and highlight the intriguing possibility that processes leading to the association and/or dissociation of SAA with HDL may represent a mechanism for modulating the biological activities of systemic SAA. Until recently, few studies have addressed how the equilibrium between lipid-free and HDL-associated SAA might be regulated. To determine whether SAA incorporates into HDL as HDL is formed, our laboratory recently investigated the lipidation of SAA by primary mouse hepatocytes, the major site of HDL biogenesis [61^••^]. We determined that SAA is efficiently lipidated by hepatocytes in an ABCA1-dependent manner to form nascent particles that are distinct from apoA-I-containing particles, indicating that SAA is not incorporated into HDL during HDL biogenesis. Our results are in line with earlier studies [36, 62, 63]. The finding that the initial lipidation of SAA does not give rise to particles containing both apoA-I and SAA raises questions as to how SAA associates with apoA-I-containing HDL in vivo. It is known that lipid-free SAA can be incorporated into HDL particles ex vivo, leading to displacement of apoA-I from HDL [63]. Whether lipidated SAA species generated by ABCA1 efficiently incorporate into mature HDL particles in the circulation, and whether this leads to the dissociation of apoA-I, requires further investigation.

While the vast majority of circulating SAA is normally found associated with HDL, a small fraction of lipid-free SAA exists in equilibrium. Factors that modulate this equilibrium and facilitate the liberation of lipid-free SAA might be expected to regulate the bioactivity of circulating SAA. HDL in the circulation is acted on by a number of remodeling factors, including lipases and lipid transfer proteins that alter the lipid and protein composition of the HDL particle. Several of these factors have been shown to destabilize the HDL particle, leading to the release of lipid-poor APOA1 [64–66]. One such factor is cholesteryl ester transfer protein (CETP), which facilitates the exchange of triglycerides on triglyceride-enriched lipoproteins with cholesteryl ester on HDL. We have shown that CETP-mediated remodeling of HDL facilitates the release of lipid-poor SAA from HDL [30••, 67] as well as the transfer of SAA from HDL to apoB-containing lipoproteins [30^••^]. We further showed that the presence of SAA on apoB-containing lipoproteins was associated with increased binding to vascular proteoglycans. Thus, SAA is an exchangeable HDL apolipoprotein, and factors that enhance SAA exchange may have functional consequences by increasing the amount of bioactive lipid-free SAA and by enhancing the atherogenicity of apoB-containing lipoproteins. Interestingly, SAA has been documented to be on apoB-containing lipoproteins, especially LDL, in human subpopulations known to be at increased risk for cardiovascular disease despite the absence of elevated LDL [68–70].

Additional mechanisms for liberating SAA from HDL have been documented. For example, Gursky and colleagues demonstrated that mild oxidation of SAA-enriched HDL liberates lipid-poor/free SAA [47]. Our group reported that hydrolysis by Group IIa secretory phospholipase A_2_ also leads to SAA release from HDL [67]. Whether oxidative modification or phospholipase A_2_ remodeling of SAA-enriched HDL leads to the release of biologically active SAA has not been investigated and may be particularly relevant to the oxidative and lipolytic environment characteristic of atherosclerotic plaques and other sites of tissue injury. Another potential mechanism for liberating HDL-associated SAA in damaged tissue is through interactions with specific components of the extracellular matrix, which is known to occur during amyloid formation [71]. HDL remodeling by phospholipid transfer protein (PLTP) and cholesteryl ester uptake mediated by scavenger receptor B1 (SR-B1) have been documented to generate lipid-poor APOA1 [64–66]. Whether the action of PLTP or SR-BI also leads to the liberation of lipid-poor SAA from HDL has not been reported.

While the liver is the major source of circulating SAA during an acute phase response, SAA is also expressed in many non-hepatic tissues, including the intestine, adipose tissue, kidney, and lung, among others [72]. The lipidation state of locally produced SAA within a tissue microenvironment is not known. One possibility is that SAA in extra-hepatic tissues exists in an oligomeric form and changes in the organization of SAA oligomers influence its bioactivity. This scenario is suggested by the elegant work of Smole et al., who recently investigated the role of SAA in asthma pathogenesis [73^••^]. The authors provide a model, whereby SAA1 produced by airway epithelial cells is activated through its interaction with dust mite allergen, which triggers the dissociation of biologically inactive SAA1 hexamers, leading to the generation of bioactive SAA capable of stimulating type 2 immune responses. Other recent studies highlight a role for SAA in both homeostatic functions and pathogenic, pro-inflammatory functions, depending on its tissue source. For example, in the gut, ileum epithelial cell-derived SAA1/2 is thought to support barrier integrity by orchestrating homeostatic Th17 cell responses [74]. On the other hand, systemic SAA is also thought to promote pathogenic Th17 programming in models of colitis and experimental autoimmune encephalomyelitis [16^••^]. Clearly, we are just beginning to appreciate the complex interplay between systemic liver-derived SAA that is induced during an acute phase response and the locally produced SAA present in tissues under homeostatic and/or inflammatory conditions.

## Conclusions

While earlier research focused on the impact of liver-derived SAA on HDL function during an acute inflammatory response, more recent studies highlight how HDL profoundly influences the function of SAA. To fully understand SAA biology, a more complete understanding of the factors that modulate SAA’s association with HDL is needed. During an acute phase response, HDL may serve as a vehicle to transport SAA to sites of tissue injury but, in the circulation, sequesters SAA to protect the host from unrestrained inflammation and generalized tissue damage [30••, 75]. Given that HDL-associated SAA appears to be biologically inert, a fundamental question is how SAA might be liberated from HDL to exert pro-inflammatory effects during an acute phase response. Understanding the mechanisms that influence the equilibrium between HDL-associated SAA and other forms of SAA may provide insights into factors leading to SAA-driven disease in individuals with chronic inflammation. Another important aspect of SAA that merits future investigation is the interplay between systemic, liver-derived SAA and locally produced SAA that is present in non-hepatic tissues under both homeostatic and inflammatory conditions. It was recognized more than 50 years ago that one pathological consequence of SAA’s dissociation from HDL is the formation of amyloid deposits in tissues of certain individuals with chronic or recurrent inflammation. Understanding the features of tissue microenvironments that facilitate the disassociation of SAA from HDL may provide insights into SAA’s pathological effects in chronic diseases, including atherosclerosis, abdominal aortic aneurysms, and cancer. The recent link between SAA and an emerging infectious disease, COVID-19 [76, 77], underscores the importance of understanding SAA’s bioactivities in both acute and chronic inflammation. Future research will uncover new insights into this ancient molecule and how it contributes to modern maladies.
